# Current care for victims of sexual violence and future sexual assault care centres in Belgium: the perspective of victims

**DOI:** 10.1186/s12914-019-0207-5

**Published:** 2019-06-27

**Authors:** Laura Peeters, Anke Vandenberghe, Bavo Hendriks, Christine Gilles, Kristien Roelens, Ines Keygnaert

**Affiliations:** 1 International Centre for Reproductive Health (ICRH), Department of Public Health and Primary Care, Faculty of Medicine and Health Sciences, Ghent University, Corneel Heymanslaan 10, UZP114, B-9000 Ghent, Belgium; 2 Department of obstetrics and gynecology, CHU Saint Pierre, Université Libre de Bruxelles, rue Haute 320, 1000 Brussels, Belgium

**Keywords:** Sexual violence, Rape, Victims, Belgium, Healthcare Centre

## Abstract

**Background:**

Sexual violence is a global health problem. After ratifying the Convention of Istanbul in 2016, this Belgian study was set up to map the perspective of victims of rape on the current sexual violence care provision in Belgium and to inquire on their need for more specialised and holistic care in future Sexual Assault Care Centres.

**Methods:**

Sixteen rape victims participated in this sub-study. A mixed-method design (questionnaire, in-depth interview or small focus group) was applied depending on the time elapsed between rape and participation. Descriptive Thematic Framework Analysis was performed in duo.

**Results:**

The participants thought it of utmost importance that every victim should receive all medical, psychological and forensic care without necessarily having to involve the police first. They stated that the current Belgian sexual violence care provision could be much more patient-centred, specifically the forensic examination and psychological care. Alongside medical and psychological consequences, victims emphasised the high personal financial and relational burden of sexual violence.

The holistic care offered in Sexual Assault Care Centres was perceived to enhance the recovery process of victims of sexual violence. Their doors should be open to all victims and their relatives. They should not only provide acute care for the victim, but also improve victims’ reintegration into society while reducing their personal costs significantly.

**Conclusion:**

All care for victims of sexual violence, especially forensic and psychological care, needs drastic improvement in Belgium. All participants agreed that having specialised, multidisciplinary and longitudinal care in a Sexual Assault Care Centre that would be open 24/7 for everyone, victims and their significant others, would be an improvement to the currently available care all over Belgium.

**Trial registration:**

This research was registered on April 1st 2016. Registration number B670201628242.

## Background

### Definition of sexual violence

The World Health Organization defines sexual violence (SV) as ‘Any sexual act that is perpetrated against someone’s will, committed by any person regardless of their relationship to the victim, in any setting. It includes but is not limited to rape, attempted rape and sexual slavery, as well as unwanted touching, threatened sexual violence and verbal sexual harassment’ [1].

### The Belgian situation

In Belgium, the Penal Code distinguishes between voyeurism, indecent assault and rape. The latter is defined as any act of sexual penetration of any kind and by any means committed to a person in absence of consent [2]. A population-based study from 2012 claimed that in the Flemish region of Belgium, 22.3% of girls and 10.7% of boys were victims of SV before the age of 18. In terms of the population over 18, 13.8% of women and 2.4% of men were victimised [3]. A multi-level analysis in 10 European countries reported that 20.4% of women and 10.1% of men aged between 18 and 27 years were victim of SV in Belgium [4]. For minorities those results were even higher with 41.1% of Flemish people who identify as lesbian, gay or bisexual (LGB) [5], 31.7% of Belgian transgender people [6] and 21,1% of migrants [7] reporting having been sexually victimised in Belgium.

Two other sub-studies mapped the current existing Belgian health services for victims of SV. On the one hand they noticed a clear fragmentation of existing health services which is a barrier in care accessibility [8]. On the other hand they noted that the Belgian hospitals are adequately equipped according to the international guidelines but a lack of cooperation between the different services, a lack of standardised protocols, and insufficient training of caregivers inhibit the expected provision of an integrated and coordinated care [9].

### Consequences of sexual violence

SV can have multiple and prolonged consequences on different levels. There are physical, psychological, relational and financial consequences. Possible physical consequences include injury, Sexually Transmittable Infections (STIs) and unwanted pregnancy [10]. Furthermore, a wide range of possible psychological consequences has been frequently reported, such as anxiety, depression and suicidal ideation [11–13]. Rape has been found to be the trauma most associated with Post-Traumatic Stress Disorder (PTSD) in women [14]. 30.2–39% of female victims of rape are being diagnosed with the disorder in the months following the event [15, 16]. Having a history of victimisation and already having used mental healthcare are generally considered as risk factors for developing PTSD [17]. Being diagnosed with PTSD also raises the chances of future drug addiction and other psychiatric disorders [18], as well as revictimisation [19]. Relationships between a victim of SV and its entourage can be strained due to the violence [20] which can lead to social isolation [21]. Importantly, the financial consequences of SV should be taken into account. There are substantial costs associated with the needed care after the violence [22] and additional costs incur indirectly due to absenteeism, self-care and others [20]. Finally, being a victim of SV raises the risk of revictimisation [23].

### Care needs of victims of sexual violence according to international guidelines

International guidelines indicate that a victim of SV should receive forensic, medical and psychological care in the acute phase as well as adequate medical and psychological follow-up care in the years following upon the violence [12, 24, 25]. In order to collect DNA from the assailant, it is recommended that the forensic examination is performed as soon as possible using a systematic, standardised protocol [26, 27]. In Belgium, this was formerly known as the “Sexual Aggression Set (SAS)”. According to current Belgian law, a victim of SV has to press charges in order to have a SAS administered [28]. The vaginal examination (including vaginal rinsing) is performed with a speculum [24]. Additionally, pictures are to be taken of possible injuries [29].

The medical care mainly focuses on treatment of injuries and prevention of STDs and unwanted pregnancies. Combining the acute medical care with the forensic examination is proven to be less traumatic for the victim [24]. It has been advised to carry out a mental state examination at first encounter [30], but it is especially important to make sure the victim knows where to go if he or she needs help during the period following the SV [24].

Guidelines further recommend regular medical and psychological follow-up sessions to assure optimal care [24, 25]. The National Institute for Health and Care Excellence (NICE) guidelines advise to perform watchful waiting on every victim of SV during the period of 1 month. During this month it is important to closely observe and assist the victim without necessarily actively treating him or her. This is because not every victim will show symptoms of PTSD in the immediate time following the sexual violence but might start to show symptoms in the month following the traumatic event [12]. If a victim is diagnosed with PTSD, adequate and specialist care and therapy must be implemented. Cognitive Behavioural Therapy (CBT) and Eye Movement Desensitisation and Reprocessing therapy (EMDR) have both proved to be effective treatments for PTSD symptoms upon SV [12, 31].

### The situation abroad

Since the Convention of Istanbul, 46 countries signed a treaty to prevent and combat SV [32]. A few neighbouring countries already introduced Sexual Assault Care Centres (SACC) into their healthcare system, e.g. “*Centra voor Seksueel Geweld”* in the Netherlands [33], Sexual Assault Reference Centres [34, 35] and Rape Crisis Centres [35] in the United Kingdom and Sexual Assault Treatment Units in Ireland [26]. Other types of centralised care for victims of SV include: Sexual Assault Response Teams [36] and Sexual Assault Nurse Examiners (SANE) [37] in the United States and Centres for Rape Victims in Denmark and Sweden [38].

### Hypothesis: the sexual assault care Centre (SACC) in Belgium

Based on experiences from those centres abroad, a theoretically ideal SACC for Belgium was designed as a hypothesis: The hospital-based centre would enrol a SANE as key caregiver for all acute forensic, medical and psychological care needs, supported by a multidisciplinary team of health professionals. Psychological, medical and social follow-up care would be closely monitored by a case-manager for at least one and up to 12 months. If the patient wishes to file a complaint, it is possible to do so up until 6 months after their initial visit at the SACC. If needed, the victim’s relatives could receive psychological support in the SACC. Contact with fellow victims would also be organised in a supervised setting [39].

### Research objective

The objective of this study was to inquire deeply on former victims’ evaluation of the currently provided care in Belgium and to obtain their opinion on the hypothesis mentioned above in order to take their experience and knowledge into account when designing a model fit for the Belgian healthcare context for pilot testing.

## Methods

### Sampling phase

The study method was twofold. On the one hand victims who just received a SAS were asked about their evaluation of the received care through a questionnaire provided at the treating hospital. The questionnaires consisted of a part A to be completed as soon as possible after having the SAS administered and a part B to be completed 1 month later. The recruitment of victims who just received a SAS took place in two Belgian hospitals: the University Hospital of Ghent and the University Medical Centre of Saint Peter in Brussels. These hospitals were chosen because of their advanced procedures for the assessment of victims of SV.

Every victim of SV above the age of legal consent to sex in Belgium (16 years old) who came in for a SAS kit between May and November 2016, should have received a closed envelope with an informational letter, an informed consent form and a questionnaire in Dutch or French. In practice, this did not always happen. Given reasons consisted of forgetfulness of the doctor who was responsible for the SAS, or ill-informed doctors. In the University Hospital of Ghent, a total of 35 victims of SV came in for a SAS kit between May and November 2016. Nineteen of them received an envelope. In the University Medical Centre of Saint Peter in Brussels, a total of 57 victims of SV came in for a SAS kit between May and November 2016. Thirty-two of them received an envelope. Of those 51 envelopes that were handed out, two completed Dutch questionnaires and a part A of a French questionnaire were collected.

On the other hand, former victims were questioned by means of an individual in-depth interview or in small focus groups. The recruitment was organised via an online invitation posted on relevant Belgian websites and discussion fora for SV victims (*Sensoa, Wij Spreken Voor Onszelf,* ICRH*, slachtofferhulp (CAW), slachtofferonthaal en slachtofferbejegening, Çavaria,* *seksueelmisbruik.info**, transgender infopunt* and *SOS Viol*), as well as disseminated through existing victim support networks. The only inclusion criteria used for this group were being a victim of SV, having sought care for it and being older than 16 years old. They could choose to participate in either an individual in-depth interview or a small focus group with several victims of the same gender. Interviews were conducted from June until November 2016. This was the limitation date given by the ethical committee and deadlines of the study itself and proved just sufficient time to obtain a certain degree of saturation in the given answers.

Twenty participants (six men and fourteen women) responded to the online invitation by electronically contacting the study coordinator to make an appointment for an in-depth interview or a focus group. Making an appointment appeared not to be easy and rescheduling was done regularly. Seven participants first confirmed their eagerness to participate but eventually did not make it to their appointment. Two of them gave bad timing (e.g. exams, holidays) as a reason for their absence. A third one received negative advice from his doctor to participate. A total of 13 former victims (9 women and 4 men) eventually participated in nine in-depth interviews (six in Dutch and three in French) and two small focus groups (both Dutch) with two participants every time. They were held in either a university meeting room at the university hospital site or in an office of a care organisation. One or two researchers were present and the interview was recorded with a voice recorder. The average time needed for an in-depth interview was two hours and for a focus group two hours and 40 min.

Every participant had the opportunity to tell us about his or her experience with police and health services they sought upon victimisation and to explain the short and long term consequences of their sexual victimisation on a physical, psychological, financial and relational level. They were also asked to give their opinion on and advice for the future SACC. The interview and focus group guides were drafted in such a way that the participant could emphasise whatever he or she perceived as important, which made every interview and focus group dynamic and unique.

The participants were not induced to participate in the study. Yet, once having completed the in-depth interview or focus group, the participants received a gift voucher (value of €15) to compensate their time spent. They did not know they would receive this when they volunteered to participate and it was meant as a compensation for possible transport costs.

### Analysis phase

The questionnaires were digitalised and the interviews and focus groups were typed *ad verbatim*. All socio-demographic quantitative results were grouped in an SPSS database. The qualitative results were grouped in an NVivo database. Before coding started, a basic code tree based on the questions in the interview and focus group guide was constructed. During coding, this tree was adapted based on the results of the Descriptive Thematic Framework Analysis where similarities and differences were sought in the answers of the different participants in duo.

## Results

### Profile of the participants

Table 1 presents the demographic characteristics of the participants. A total of sixteen rape victims participated in the study. The average age of the participants was 33 years and 4 months. The median was 34 years and 6 months. The youngest participant was 17, the oldest 61 years old.

**Table 1:** Profile of participants (*N* = 16)

Gender	N
Male	4
Female	12
Age	
< 20	2
20–29	4
30–39	8
40–49	1
50–59	0
60–69	1
Sexual orientation	
Heterosexual	11
Homosexual	1
Bisexual	1
Other^a^	3
Country of origin	
Belgium	14
Spain	1
Algeria	1
Civil status	
With parents and no partner	1
With parents and partner	1
Living alone and no partner	8
Living alone and partner	1
Living with partner	5
Children	
No children	12
Children always with them	3
Children part-time with them	1
Attained education	
Primary school	2
Secondary school	3
Higher education (not university)	3
University	8
Daily activities	
Student	5
Working	9
Other^b^	2
Native language of the participant	
Dutch	12
French	2
Spanish	1
Arabic	1

^a^Other sexual orientations were pansexual (one participant) and ‘questioning’ (two participants)

^b^One participant is admitted but did not specify where. Another one is training to become an art therapist

With the exception of participant number 6, all participants were already exposed to violence prior to the physical sexual violence which was at the core of the interview. For twelve of them this regarded sexual innuendo and groping, while nine recounted to have been a victim of prior verbal abuse as well as physical threats. This prior violence was defined as: verbal harassment, physical abuse or sexual violence with or without penetration by any person.

Thirteen participants told us about their assailant. They were all men. Eight participants knew their assailant before the violence; the remaining five met their assailant for the first time on the day of the violence. Seven participants were minors at the moment of the rape.

### Perspective on the current situation in Belgium

After the SV, the participants had sought assistance at various services including the police, hospitals, general practitioners and psychologists. Their experience with these services will be discussed in that order underneath. Furthermore, the consequences of their SV and their opinion on future SACC were discussed. The results will be described as closely as possible to the answers given by the participants. Illustrative quotes were added.

#### The police services

Seven participants pressed charges immediately after the violence. Five other participants did so after a while (the longest period being 45 years). Their opinion on this experience of pressing charges was dependent on the approach of the individual police officer who took note of their complaint.

*“I did not want to press charges but they forced me. The police officer was a monster. A horrible woman who just wanted to close her case so she could go home. We stayed there until 3 AM. I yelled, I cried, I said I did not want to, but she threatened me so in the end I did it. She was violent and it was horrible.” (P14, woman, 20-29yo).*

*“That was the first time I told my story and she didn’t judge me. That was a very good first experience.” (P4, woman, 30-39yo).*

Regardless of the gender of the participants, all stressed the importance of the gender of the police officer, emphasising they would prefer to see a woman rather than a man in this situation. Yet, one participant did reason that only implying female officers would make the matter a gender specific problem.

*“I can only say for myself that I would feel more comfortable with a female caretaker.” (P15, man, 60-69yo).*

*“If you only ever talk about the matter with women and female police officers, men will never be able to hear about it and they will never understand what’s wrong with other men. I understand you need a safe space but otherwise it just becomes a women’s problem.” (P8, woman, 20-29yo).*

An important point perceived by the participants was separating the needed healthcare after SV from pressing charges. A victim of SV should be able to receive all forensic, medical and psychological care without being forced to file a complaint. This enables the victim to press charges whenever they feel ready in order to make filing a complaint part of the healing process rather than compounding the damage.

*“It is very important in the recovery of a person that filing a complaint is done at the correct time to create a feeling of victory. (...) First recovery, then prosecution.” (P10, man, 30-39yo).*

A last aspect was the period of legal limitation. Since November 2011, this period equals 15 years in Belgium. Four participants found that this limitation time is too short.

*“Having no limitation period might be utopic, but if that would be possible, that would be great.” (P5, woman, 30-39yo).*

#### Care received at a hospital

Ten participants sought medical care immediately upon the victimisation. Six of them had agreed to have a SAS administered but only two of them told us specifically about this experience. Both perceived the experience as unpleasant, wishing it would be over as soon as possible.

*“I just counted the seconds until I was allowed to put my clothes back on.” (P2, woman, 30-39yo).*

Two participants specified further that using a speculum at that time could best be avoided.

Four participants accepted the psychological assistance provided by the hospital. Two of them appreciated the assistance while the other two did not. All of them thought that the assistance afterwards, when provided, ended too soon.

*“It (assistance) would be good because I did not speak to anyone for three weeks afterwards.” (P12, woman, 30-39yo).*

#### Care received from a general practitioner

Five participants went to their general practitioner in their search for help after the victimisation. Two participants did this immediately upon the victimisation. Three of them were satisfied with the provided care, while the remaining two were not, relating it to knowledge and skills as:

*“It did not seem like he felt comfortable with the situation. (...) He did not refer me nor did he examine me.” (P16, woman, 30-39yo).*

#### Psychological care

Psychiatric care, provided by a physician with a degree in psychiatry, is reimbursed in Belgium, but psychological care provided by a psychologist is not yet reimbursed. However, free psychological care can be obtained through various organisations if you match certain criteria. There are psychiatrists and clinical psychologists that have a university degree and are specialised in the matter, but there are also so called ‘therapists’ who do not have that university degree but are still able to consult because the title of therapist is not legally defined in Belgium. This difficulty to find educated therapist was addressed by different participants. As an example one participant told us about ‘teddy bear therapy’, where participants lay on each other believing human warmth would help to process their trauma.

*“You had to lie down on a mattress and then someone lay down on top of you as a sort of teddy bear to make you feel safe so your own feelings could surface more easily.” (P15, man, 60-69yo).*

Thirteen participants told us about their search for adequate psychological care. One participant told us she had been looking for psychological care but was unable to find it.

*“I never found a place where someone told me: Listen, we are going to treat the fact that you were raped. Never.*” *(P14, woman, 20-29yo).*

Twelve other participants did seek and found psychological care. In the majority of the cases this help came from more than one caregiver.

Six participants sought free psychological care but only one felt like this free care was sufficient. Eleven participants resorted to the services of a paid healthcare practitioner (six with a psychologist, four with a psychiatrist, six did not specify). Four participants were still in therapy at the time of the interview. Two participants found help in alternative methods, one in spirituality and the other in mindfulness and Buddhism.

*“That’s working on me on another level than therapeutic. It is a relief that I can work on this not only in therapeutic context, that there are other possibilities.” (P5, woman, 30-39yo).*

Five participants were tested on PTSD and all of them tested positive.

### Consequences of the sexual violence

We asked all participants to evaluate their general health prior to the violence and at the current moment. The results can be found in Table 2.

**Table 2:** Subjective health rate prior to and after the sexual victimisation (*N* = 16)

Subjective health prior to the sexual violence	N	Subjective health after the sexual violence	N
Very good	10	Very good	4
Fairly good	2	Fairly good	6
Not good, not bad	1	Not good, not bad	2
Fairly bad	0	Fairly bad	1
Very bad	1	Very bad	2
Missing	2	Missing	1

While interpreting these results, the lapse between the violence and the interview needs to be considered. The shortest interval was 3 days, the longest 47 years. The average time was 10 years and 2 months. Nine participants feel worse now than before the violence. Only one participant regarded himself as being in better health now than before the violence. Four participants saw no change in their health.

Eleven participants emphasised that they still suffer from the consequences of the SV. Those consequences were primordially psychological (ten participants) with mostly PTSD, or PTSD symptoms being described.

*“I still cry. I try to understand why it happened. I feel sad. Sometimes I’m aggressive. My reactions are more radical. It is like I’m always on my nerves.” (P12, woman, 30-39yo).*

Five participants still suffered on a physical level with mostly sleeping disorders being disclosed. Four participants still suffer on a relational level, feeling socially isolated.

*“A full pub, events with a lot of noise… those are difficult situations. Especially right after the violence. Those emotions are not understood by my environment.” (P16, woman, 30-39yo).*

#### Financial consequences

Ten participants told us about the costs they faced upon SV. The general opinion was that the cost can be very high due to a lot of different aspects, for example acute and follow-up healthcare, legal costs, absenteeism, changes made to a room or a person’s appearance and so on.

Five of these ten participants, told us that they received less therapeutic care than they had wanted anticipated or received the care later than they had wanted due to the high cost of this care. As psychotherapy was not yet reimbursed in Belgium and with the cost of one session being 40 to 50 euros on average, this therapy could cost the victim up to three to four thousand euros per year.

*“They (the government) need to clear money (for the reimbursement of psychological care). (...) If they do not do that, they’re being stupid. (...)They are forgetting that if a survivor is not helped or is helped too late, that he or she will develop more than only psychological problems. He or she will also develop serious physical problems.” (P9, man, 40-49yo).*

#### Relational consequences

All participants who had a relationship with a significant other noticed a difference after the SV. Three went to a relationship counsellor. In two cases this helped the participant to find a *modus vivendi* with their partner. One participant was not helped by the counselling and she felt her relationship was falling apart at the time of the interview.

*“Cracks have been forming in the relationship between my boyfriend and I and it would be a shame if that would be lost as well. (...) A friend psychiatrist told me that the partner of a victim of sexual violence is also traumatised and should also be able to get his side of the story off his chest but I do not think my boyfriend is in a place where he can accept that he has been traumatised as well.” (P16, woman, 30-39yo).*

Two participants saw a relationship end due to the SV, as their partner did not react appropriately and did not give them support. In contrast, two participants did receive support from their partner and as a result, recalled it making their relationship stronger.

*“He was unable to understand that someone had done that to me. It was my fault. It was really catastrophic. He had no empathy whatsoever.” (P3, woman, 30-39yo).*

*“The violence certainly had an influence on our relationship, but I believe it made us stronger. He understands that I can suddenly react very emotionally.” (P2, woman, 30-39yo).*

Eleven participants confided in a family member about the victimisation. In general it could be observed that if the confidant responded adequately, their relationship grew stronger. In case the confidant responded by blaming or not believing the victim, their relationship suffered damage. Neither of the two participants who were not Belgian nationals spoke to their family about the event, believing this was impossible.

Four participants are parents. In three cases, their children played an important role in their process of what happened to them. Two participants started therapy at the birth of their son.

*“The main motivation I still have for therapy is to be a better father.” (P10, man, 30-39yo).*

One participant already had kids before the violence. They were her reason to never give up.

*“Without knowing so, they ensured that I kept going. They are the reason that I am still here at this point.” (P2, woman, 30-39yo).*

The same applies to relationships with friends as to the relationship with a family member. If the friend reacted in a positive way, with empathy and by supporting and believing the victim, their relationship grew stronger. But sometimes friends did not react appropriately. Participants told us they lost friends because of the violence, which, in some cases, led to social isolation.

### Perspective on sexual assault care Centres

All participants reacted positively to the potential establishment of SACCs. Centralising the currently fragmented care, not having to repeat their story time after time and knowing where to go for specialised care were all seen as strengths of the future SACCs.

*“That everything would be centralised, that would be fantastic. Otherwise you are totally lost. You do not know what to do, how to react, where to go.” (P12, woman, 30-39yo).*

*“Centralise everything so you do not have to repeat everything fifteen times and you do not have to wait for a long time at different places so you can go home faster.” (P6, woman, <20yo).*

#### Tasks the SACC should fulfil

All participants thought it to be logical that all acute care (forensic, medical and psychological) would be available in the centres. Adequate psychological care consisted of more than a single contact in their opinion. Furthermore, raising awareness within society was mentioned nine times.

*“Education at school and at home is necessary because I think there are men for whom it is not sexual assault. (...) You are in the street so I am allowed to touch you. (...) It is the rape culture and it is normal.” (P12, woman, 30-39yo).*

Raising awareness should be twofold. On the one hand it is necessary to educate the society as a whole in order to prevent SV. On the other hand people need to learn how to react when a person close to them becomes a victim of SV. This should ensure optimal care for the victim and prevent social isolation.

Furthermore three participants recommend legal advice to be given in the SACC, three would like addiction treatment, two would like a sexologist and two would like a relationship mediator to be present in the centres.

*“Working on future addictions is also important because people are going to try and cure themselves from their fear. (...) Try to channel future self-medication.” (P3, woman, 30-39yo).*

When given the choice, all participants –including the male ones– would choose a woman as caregiver. However, three participants believed that learning to trust a man again is an important step in the healing process.

*“I think it depends where you stand in the healing process. I know I can’t stay calm when a man, especially when he’s standing behind me, touches me. But I think further down the road this could be possible. Plus the fact that 10% of assailants are women. So it is not necessarily positive to take a female caregiver as the standard.” (P10, man, 30-39yo).*

*“A man would be a problem I think.” (P12, woman, 30-39yo).*

*“It is twofold because it would be healing to be able to trust a man on the issue.” (P5, woman, 30-39yo)*

Contact between fellow victims was discussed in all conversations except one. Three participants did not see the appeal of a support group. Nine others told us they had searched for support groups. In their opinion the feeling of being normal and understood could only be offered by people who had lived through the same traumatic experience.

*“Something I missed at the start of my healing process was contact with fellow victims. Because I was wondering if how I reacted was normal.” (P9, man, 40-49yo).*

When discussing the accessibility of such a centre, transportation to and from the centre should be possible according to two participants. Additionally, four participants think the centre should be accessible via phone or a chat room. Three participants would recommend the possibility to stay the night if the victim did not feel safe at home.

*“Both (phone and chat) are necessary because sometimes you do not have the force to speak, sometimes you do not have the force to type.” (P14, woman, 20-29yo).*

*“The distance created by a screen and the fact that there’s nobody in front of you that can touch you can be necessary. (...) Sexual violence is mostly present in the age group that was raised with social media and is stuck on their screen 24/7. So it is logical for the government to respond to that.” (P10, man, 30-39yo).*

A last point was the stage setting of the centre. Four participants thought the centre should not be too clinical because they are not sick, but traumatised. They need to feel safe. A centre which offers more than just care could also help to make victims feel safe as suggested by one participant who said a multipurpose hall would be a good idea to organise parties and get-togethers for victims as a way to learn to reintegrate into society.

#### For whom

All participants thought that all victims should be able to receive help in the SACC. Victims of recent SV as well as former victims and their entourage should be welcomed in due fashion.

#### The name of the Centre

Finding an agreement on the name was no easy procedure. Suggested names were: Centre after sexual violence, Referral centre after sexual violence, Shelter/rescue centre after sexual violence, Care centre after sexual violence and Care centre for victims after sexual violence. The name Care Centre after Sexual Violence got the most votes with a total of three participants voting for this name, but only seven of the participants voted for one of the suggested names. The other participants perceived the suggested names as too clinical. Seven participants criticised the integration of the term ‘sexual violence’ in the name which, in their opinion, would raise the threshold to enter the centre. Five participants preferred the term ‘survivor’ to ‘victim’. Mostly, they wanted a softer name, a name that would make them feel safe. They suggested alternatives: Survivors institute, Help after coercion, the house of sexuality or a symbolic name, for example ‘The salt circle’.

*“All those names represent the negative point of view. It does not represent all the possibilities that can be done before or after the violence. All that can be done for children, in school, … [..] For example the house of sexuality. Something positive and open. [..] The most important part is that victims want to go there.” (P14, woman, 20-29yo).*

## Discussion

Three findings stand out when observing the answers given by the participants. First they agree that the judicial procedure –starting with filing a complaint- should be disconnected from the provision of medical, forensic and psychosocial care after SV. Second is the fact that they all had to search to find adequate (psychological) help, which turned out to be extremely expensive. Finally, they are unanimously positive about the idea of having a SACC instead of the current provided care.

### Profile of the participants

The number of participants in this study is too small to extrapolate conclusions for Belgium or other countries. Nevertheless, three facts stand out on the profile of the participants. Firstly, as is shown in former literature [23] every victim knew their assailant before the SV occurred. This still stands in contrast to the prevailing rape myth accepted in the general Belgian population; that assailants are mostly unknown by their victim. Secondly, twelve participants told us they had already been a victim of sexually inappropriate remarks and groping before the most recent episode of SV. There is thus still a very high acceptance of harassment and ‘lighter’ forms of SV in Belgium which is also in line with recent literature [3, 5–7] yet at societal level problematic. Thirdly and lastly, the fact that seven participants were minors at the time of their (first) sexual victimisation and four participants were male victims. This last fact emphasises the need for adequate and specialised care for minors as well as adults, both male and female, which is also important in the prevention of revictimisation [23].

### Consequences of the victimisation and current provided care

The high frequency of many health problems listed by the participants coincides with the variety of consequences listed in the literature [10–22]. This high number of adverse health consequences exists despite the sometimes long period of time between the interview and the violence, underlining the importance of long-term follow-up care.

PTSD is the most frequently described psychological consequence by the participants. Yet, it should be noted that, when asked during the interview if they had been tested on PTSD, only half of the participants responded confirmatively. Given that rape has been found to be the trauma most associated with Post-Traumatic Stress Disorder (PTSD) in women [14], it would be a logical step to test every victim of SV but this is clearly not yet the case in Belgium.

Half of the participants who answered the question told us they wanted more psychotherapy or wanted it sooner but did not pursue these courses due to the high costs, deteriorating the accessibility. Earlier literature confirms this [20, 22]. An extra difficulty exists for victims who are minors or students who financially depend on their parents but do not necessarily wish to confide in them about needing help. Adequate measures should be taken to ensure the financial accessibility of the needed care.

Jina et al. stated the relationship between a victim of SV and his entourage can suffer post assault [20]. Assessing the different responses in this study, the relational consequences can be divided into two groups. If the confidant reacted adequately, the relationship between the victim and confidant grew stronger. If not, the relationship was damaged with some of them ending. Almost all participants were confronted with both situations depending on the reaction of the person in which they confided. A victim of SV needs support during the whole healing process, so social isolation should be avoided [21]. Neither of the two non-Belgian participants told their families what had happened and emphasised it as culturally unacceptable. The cultural background of a victim should therefore be considered. This is an important factor to take into account when developing SACCs in a multicultural environment or in a country with a different culture then the Belgian culture. Therefore, the caregiver’s cultural competence and understanding are vital.

In addition to this, it is important that the relatives of a victim are taught how to interact with the victim, highlighting the need to raise awareness in the general population and the close entourage of victims. Furthermore, it is necessary to provide support in the SACC for relatives should they have questions or need help themselves as is already the case in the Netherlands [33].

### Perspective on the current situation in Belgium

#### The police services

As shown before, up until 2016 a victim needed to officially press charges against the alleged assailant in order to be able to have a forensic examination through a SAS kit in Belgium. Those were mostly administered in hospitals or forensic institutes [28]. This thus represents a predominantly medico-legal approach to SV: the body of the victim is above all considered as part of the crime scene as there is evidence to be found in and on it. The fact that it regards a person who just went through a traumatic event and needs assistance and care is often only considered at a later stage – if considered at all. All participants considered it vital that the necessity to file a complaint prior to having the opportunity for a forensic examination should be dropped. They preferred the option to have a forensic examination and/or appropriate medical care immediately and to have more time to decide whether to file a complaint when ready. This is already possible in Ireland [26], Denmark [38] the United Kingdom [34] and the Netherlands [33] where it is demonstrated that, with easily accessible information and support before, during and after the declaration, the victim would feel ready to file his or her complaint faster. Moreover the participants were in favour of having the option that the healthcare worker presses charges in case a assailant is reported more than once as is the case in Denmark [38]. After ample meetings with prosecutors and judicial experts, this has been taken into consideration in the Belgian SACC model and is tested as such in the piloting of the model.

Furthermore, it has been stressed that the police officer noting the declaration should be experienced in the delicate and traumatising subject of SV. Offering the victim the possibility to choose the officer’s gender might contribute to making the victim feel more at ease. Organising specialised training and making sure both genders of police officers are on call was put forward as a huge improvement and is now also considered for pilot testing.

#### Care received at the hospital

Optimal holistic care consist of an acute forensic, medical and psychological examination, ideally simultaneously, and medical and psychological care in the years following upon the SV [24]. The two participants who disclosed about their forensic and medical examination recalled it to be an unpleasant experience. Possible improvements are assuring that the victim is never completely naked during the examination, a human approach and clear information about the different steps of the examination. Replacing the speculum with smears would also be a step in the right direction. Those measures are all very obtainable and should be implemented immediately both in the current SAS as in the SACCs.

The opinions on acute psychosocial assistance, provided at the hospital, were mixed. The consequences victims continue to suffer from to this day, are primarily psychological [11–13]. Therefore the psychological assessment and follow-up should be optimal from the first point of contact to try and minimise the psychological consequences perceived later in life. The participants enforced the literature by saying that the presence of a safety net and a safe place to stay at discharge will help to ensure the best start of the healing process post assault [21]. This recommendation has also been taken into consideration and free psychological care comparable to the ones provided in existing SARCs will be provided in the piloting of the Belgian SACCs.

Yet, the Belgian psychological care provision needs to be improved drastically. It should be ensured that a victim of SV is treated by a clinically educated psychologist in trauma-related matters. Victims of SV are at risk of revictimisation [23]. Making sure they are correctly taken care of from the start of their healing process could avoid revictimisation and should thus be a priority of the government who subsequently needs to organise and supervise the psychological care in Belgium.

Guidelines show the best way to organise medical and psychological follow-up after SV [12, 24, 25]. The chronic care in Belgium is not organised as it should be according to these guidelines. The participants had to search for help themselves. This, again, could be harmful and possibly lead to further traumatisation if they do not find specialised care.

A last point would be the experience and gender of the caretaker. To ensure optimal care for victims from a patient-centred approach, it is primordial that all services employ experienced caretakers whose gender can ideally be chosen by the victim.

### Care for the caretaker

Hearing all those testimonials was challenging. The healthcare worker who hears those testimonials every day has no easy task at hand. Supervision should be provided in the centres to monitor the coping of the healthcare workers. This also came forward in another sub-study where caretakers asked for more supervision themselves [8]. After every in-debt interview or focus group of this study, the researchers took a moment to debrief and reflect on the testimonies to enable everyone to process the information.

### Perspective on sexual assault care centres

The participants perceived the future SACC in about the same way as put forward in our hypothesis [39]. They want a centre that provides centralised and specialist care. This would be provided by a SANE who would have four tasks. The first would be to provide all acute forensic, medical and psychological care for the victim. The second would be to make sure the victim has a safe place to go to after receiving the care he or she needed. Another task of the SANE would be to raise awareness in the entire population. A last task of the SANE would be telephone and online help. For the participants it is imperative that this help should be available round the clock, as already exists in Ireland [26] and Denmark [38]. If possible, it is recommended to allow the victim to choose the gender of the SANE.

Subsequently, the case-manager [39] will make sure there is sufficient follow-up in order to avoid the victim becoming lost in their search for help. The fact that victims of recent SV, former victims and their relatives would be welcome in the centres was crucial to all participants.

This centralised and holistic care could reduce the personal financial burden for the victim. Having SACCs will enable every victim to receive specialised care that can easily be controlled on quality and efficacy, thus making SACCs more cost-effective for the government than the present-day situation [39].

Another aspect that is part of the hypothesis is the contact with fellow victims, an aspect participants had searched for themselves in the time following their SV. This contact could be organised online or in the SACC. Anyway, it is important this contact would happen in a verified fashion to prevent further traumatisation of victims who decide to participate.

This sums up what they would expect from the SACC, but only with reference to centres for acute care needs. The participants also stretched the importance of follow-up care. Furthermore, they want a centre where they are more than victims, a centre that helps them reintegrate into society. To help them achieve that goal, the participants listed other caregivers that should be on hand in the SACCs: legal advice, a sex therapist, addiction treatment and a mediator. If this is not feasible, a referral list of appropriate caregivers should be available. A multipurpose hall would also be an option to make the centre feel safer and less clinically cold.

This need to feel safe was also reflected in the disagreement about the name. Care Centre after Sexual Violence got the most votes out of the suggestions, but less than half of the participants voted on a name from the suggestions.

### International recommendation

As the centres are organised for victims of SV it seems logical to take their opinions into account and implement their advices as much as possible. Having a sub-study dedicated to victims certainly was a strong point in the development of SACCs in Belgium. It is subsequently strongly recommended that every development of future SACCs include victims’ perspectives.

### Research limitations

Of the 92 victims of recent SV who came in for a SAS in one of the two participating hospitals, 51 received an envelope with the questionnaire and three participated in this research. Of the former victims of SV who saw our online invitation, twenty reached out to us and thirteen participated. Even though only a low percentage of reached victims participated in this study, the number of participants in this research was as would be expected for qualitative research in this field of study. Disclosure is very low for victims of SV and participating in follow-up research by victims is even lower. In qualitative research, it is expected to achieve saturation between 10 and 20 interviews. This saturation was noted in the responses given by the different participants.

## Conclusion

When inquiring on former victims’ evaluation of the currently provided care in Belgium after SV and their opinion on future SACCs the following can be concluded. Firstly, the judicial procedure needs to be disconnected from the other necessary care after SV. Secondly, all care, and especially psychological care needs drastic improvement in Belgium. A method to ensure qualified and easy-to-find clinical therapists, even to victims who cannot afford it, needs to be put in place. Thirdly, all participants were unanimously positive about the idea of a SACC. Having specialised, multidisciplinary and longitudinal care in a centralised centre that would be open 24/7 for everyone, victims and their significant others, was perceived as an improvement to the currently available care in Belgium. Those centres also have the potential to give more than only the needed care. They could raise awareness in the entire population to tackle SV as a whole. It could be a safe haven, a place where victims learn to reintegrate into society. In accordance with international examples and guidelines, it is time for Belgium, and all other countries that signed the Istanbul Convention to accept their responsibility and provide a holistic, patient-centred and sensitive care for all victims of SV.

## Data Availability

The datasets generated and/or analysed during the current study are not publicly available. They are available from the corresponding author on reasonable request.

## Abbreviations

CBT: Cognitive Behavioural Therapy
EMDR: Eye Movement Desensitisation and Reprocessing therapy
LBG: Lesbians, Gays or Bisexual
NICE: National Institute for Health and Care Excellence
PTSD: Post-Traumatic Stress Disorder
SACC: Sexual Assault Care Centre
SANE: Sexual Assault Nurse Examiner
SAS: Sexual Aggression Set
STI: Sexually Transmittable Infection
SV: Sexual Violence
